# A single-field integrated boost treatment planning technique for spot scanning proton therapy

**DOI:** 10.1186/1748-717X-9-202

**Published:** 2014-09-11

**Authors:** Xiaorong Ronald Zhu, Falk Poenisch, Heng Li, Xiaodong Zhang, Narayan Sahoo, Richard Y Wu, Xiaoqiang Li, Andrew K Lee, Eric L Chang, Seungtaek Choi, Thomas Pugh, Steven J Frank, Michael T Gillin, Anita Mahajan, David R Grosshans

**Affiliations:** Departments of Radiation Physics, The University of Texas MD Anderson Cancer Center, Unit 1150, 1515 Holcombe Boulevard, Houston, TX USA; Department of Radiation Oncology, The University of Texas MD Anderson Cancer Center, 1515 Holcombe Boulevard, Houston, TX USA

**Keywords:** Proton therapy, Spot scanning, Single-field optimization, Single field integrated boost, SFIB

## Abstract

**Purpose:**

Intensity modulated proton therapy (IMPT) plans are normally generated utilizing multiple field optimization (MFO) techniques. Similar to photon based IMRT, MFO allows for the utilization of a simultaneous integrated boost in which multiple target volumes are treated to discrete doses simultaneously, potentially improving plan quality and streamlining quality assurance and treatment delivery. However, MFO may render plans more sensitive to the physical uncertainties inherent to particle therapy. Here we present clinical examples of a single-field integrated boost (SFIB) technique for spot scanning proton therapy based on single field optimization (SFO) treatment-planning techniques.

**Methods and materials:**

We designed plans of each type for illustrative patients with central nervous system (brain and spine), prostate and head and neck malignancies. SFIB and IMPT plans were constructed to deliver multiple prescription dose levels to multiple targets using SFO or MFO, respectively. Dose and fractionation schemes were based on the current clinical practice using X-ray IMRT in our clinic. For inverse planning, dose constraints were employed to achieve the desired target coverage and normal tissue sparing. Conformality and inhomogeneity indices were calculated to quantify plan quality. We also compared the worst-case robustness of the SFIB, sequential boost SFUD, and IMPT plans.

**Results:**

The SFIB technique produced more conformal dose distributions than plans generated by sequential boost using a SFUD technique (conformality index for prescription isodose levels; 0.585 ± 0.30 vs. 0.435 ± 0.24, SFIB vs. SFUD respectively, Wilcoxon matched-pair signed rank test, p < 0.01). There was no difference in the conformality index between SFIB and IMPT plans (0.638 ± 0.27 vs. 0.633 ± 0.26, SFIB vs. IMPT, respectively). Heterogeneity between techniques was not significantly different. With respect to clinical metrics, SFIB plans proved more robust than the corresponding IMPT plans.

**Conclusions:**

SFIB technique for scanning beam proton therapy (SSPT) is now routinely employed in our clinic. The SFIB technique is a natural application of SFO and offers several advantages over SFUD, including more conformal plans, seamless treatment delivery and more efficient planning and QA. SFIB may be more robust than IMPT and has been the treatment planning technique of choice for some patients.

## Introduction

In spot scanning proton therapy (SSPT), a pencil beam (spot) is magnetically scanned in both directions lateral to the beam direction to produce a large field without the introduction of scattering elements or range modulation devices into the beam path [1–4]. Monoenergetic pencil beams of different energies are used to produce the desired depth dose distribution. The intensity of each spot can also be modulated to deliver intensity-modulated proton therapy (IMPT) [5–9]. To achieve the desired dose distribution over the target volumes, the weights of individual spots are optimized using an inverse planning process. There are two general approaches to optimizing a SSPT plan. The first is to optimize the weights of all spots in all fields simultaneously to produce the desired dose distribution. This approach is called multiple-field optimization (MFO), and is more commonly known as IMPT [5–10]. The second approach is to optimize the weights on a field-by-field basis, that is, each field is optimized individually to deliver a fraction of the prescribed doses to the entire target volume(s). This method is called single-field optimization (SFO) [11]. The most common application of SFO is to produce a uniform dose over the entire target volume by each field, known as single–field uniform dose (SFUD) [5, 11–13].

An extension of SFO techniques would allow for the incorporation of a simultaneous integrated boost (SIB), an approach which we have termed, single field integrated boost (SFIB). The concept of SIB was originally proposed for intensity modulated radiation therapy (IMRT) using photons [14] and has been widely used since. For a patient with multiple target volumes to be treated at different prescription dose levels, one could develop multiple treatment plans using SFUD to sequentially treat the patient. However, optimizing plans separately may produce inferior results when plans are combined. Moreover, additional time is required for treatment planning as well as quality assurance of individual plans. Like IMRT, IMPT can be naturally used to achieve SIB [5–9]. IMPT is considered more flexible than SFUD and often delivers more conformal dose distribution to target volumes and lower doses to critical structures. However, IMPT plans may be more sensitive to the uncertainties associated with proton therapy and thus less robust than SFUD plans [7]. Given the limitations of current commercial treatment planning systems (TPS), at the University of Texas MD Anderson Cancer Center (UTMDACC) Proton Therapy Center, we normally treat our patients with the SFO (either SFUD or SFIB) technique [13], unless the IMPT plan is significantly better than the SFO plan. In this work, we present examples of treatment planning techniques of SFIB implemented in our clinic.

## Materials and methods

### Patients and treatment planning

Since April 2010, we have employed an SFIB technique for planning and treatment of patients at the UTMDACC proton therapy center. Diseases planned and treated with this technique include prostate cancer, thoracic tumors, central nervous system tumors (brain and spinal lesions), and head and neck tumors. All patients are enrolled in Institutional Review Board approved prospective studies of proton therapy. To demonstrate the SFIB technique and qualitatively assess it’s benefits and versatility, here we present four representative clinical cases each with multiple target volumes requiring different dose levels.

We utilized SFO planning with dose constraints employed to achieve the desired coverage of different target volumes by differing dose levels within in a single plan. Target volumes treated with the SFIB technique may include a target within a target, such as for patient 1 and 4; targets located side by side, such as for patient 3; or a combination of these two situations, such as for patient 2 (see below for detailed description of each case).

SFIB treatment plans as well as sequential boost SFUD and IMPT plans were generated for comparison. Dose levels for SFUD plans were determined based on the linear quadratic model and the dose levels chosen by the treating physician for the clinically utilized SFIB plan. Doses were rounded to integer number of fractions. IMPT plans used identical dose levels as SFIB plans. Treatment plans for all patients were developed using computed tomography (CT) images obtained using 120-kV X-rays on a 16-slice scanner (LightSpeed RT 16; GE Healthcare, Waukesha, WI). In treatment planning, all proton doses were expressed as Gy(RBE) with a constant RBE factor of 1.1. The Eclipse TPS (version 8.9; Varian Medical Systems, Palo Alto, CA) with a proton module was used. Inverse planning using an SFO and MFO options with simultaneous spot optimization, using constraints for target volumes and critical structures, was used for all patients. The details of our clinical commissioning of the scanning beam dose model in TPS is described separately [15]. Each spot’s position, energy, and number of monitor units (MUs) were determined using the TPS. Beam parameters for each field are summarized in Table 1.

**Table 1:** **Field parameters**

Patient	Field	Gantry/Couch	Max/Min Energy (MeV)	Max/Min Range (cm^2^/g)	σ_air_for Max/Min Energy (cm)	Number of Energy Layers	Spot spacing (cm)
**1**	Left vertex 1	60°/290°	138.1/91.1	13.3/6.2	0.80/1.16	30	1.2
	Posterior	180°/0°	144.9/106.6	14.5/8.3	0.77/1.02	22	1.2
	Left vertex 2	95°/290°	134.6/91.9	12.7/6.3	0.82/1.16	29	1.2
**2**	Posterior	180°/0°	132.8/79.7	12.4/4.8	0.83/1.33	37	1.1
**3**	Right lateral	270°/0°	198.3/155.3	25.2/16.4	0.59/0.73	19	0.64
	Left Lateral	90°/0°	198.3/155.3	25.2/16.4	0.59/0.73	19	0.64
**4**	Right anterior	300°/15°	143.2/96.9	7.5/0.3*	0.95/1.59	32	1.0
	Right lateral	270°/15°	139.8/96.9	6.9/0.3*	0.97/1.59	30	1.0

*Abbreviations:* *Max* maximum, *Min* minimum, *E* energy, *R* range, *σ*_*air*_ sigma of spot in air at the isocenter. Spot spacing, center-to-center distance of spot. *With a range shifter of 6.7 g/cm^2^.

### Patient 1 central nervous system - brain

A young patient with a glioma of the tectal plate was treated with SSPT. The patient was immobilized using a Precise Bite positioner attached to a thermoplastic mask with a polyurethane foam headrest (CIVCO Medical Solutions, Orange City, IA). The SFUD, SFIB and IMPT plans used the same three fields: two left oblique superior vertex fields and one posterior– anterior (PA) field. For SFIB and IMPT planning, the clinically prescribed doses were 54 and 50 Gy(RBE) to the CTV1 and CTV2, respectively, in 30 fractions. The corresponding dose to CTV2 for the SFUD plan was 45 Gy(RBE) in 25 fractions, followed by a boost to 54 Gy(RBE) for CTV1. The planning target volume (PTV) was defined as a 3-mm expansion of the CTV. Strictly speaking, the PTV concept commonly used in photon therapy cannot be directly used for proton therapy because the range uncertainties are beam-direction-specific. However, beam-specific PTVs (bsPTVs) [16] are not available in our clinical TPS. We nevertheless currently use the PTV concept in spot scanning proton therapy to manage delivery uncertainties for plans with a non-parallel-opposed beam arrangements [7].

### Patient 2 central nervous system - spine

An adult patient with a myxopapillary ependymoma of the spine was treated with SSPT with a single PA field. The patient was immobilized in the supine position with a knee and foot-positioning device (CIVCO Medical Solutions, Orange City, IA). A digital model of the treatment couch with correct water-equivalent thickness was used to replace the CT couch. For SFIB planning and treatment, the prescription doses were 54, 50.4 and 45 Gy(RBE) to the CTV1, CTV2, and CTV3, respectively, in 28 fractions. The corresponding doses for the SFUD plan were calculated at 50.2 Gy(RBE) to CTV2 in 26 fractions and 42.5 Gy(RBE) for CTV3 in 22 fractions. The PTVs were defined as a 3-mm expansion of the CTVs except the anterior expansion of 5 mm to account for range uncertainty, but sparing the majority of the vertebral bodies.

### Patient 3 prostate

A gentleman with an adenocarcinoma of the prostate was treated with SSPT with two equally weighted, parallel, opposed lateral fields. The simulation and treatment planning technique for prostate patients has been described in detail previously [11, 13, 17]. PTVs defined for prostate patients may be considered as a simplified version of bsPTV [16] and used to account for range and setup uncertainties. For SFIB and IMPT planning, the desired prescription doses were 78 and 60 Gy(RBE) to the CTV1 and CTV2, respectively, in 39 fractions. The corresponding dose to CTV2 for the SFUD plan was 54 Gy(RBE) in 27 fractions, again followed by a boost to 78 Gy(RBE) to CTV1.

### Patient 4 head and neck

A patient with an acinic cell carcinoma of the right parotid was treated with SSPT. The patient was immobilized using a Precise Bite positioner attached to a thermoplastic mask with a customized head mold using RediFoam^TM^ (RediFoam, CIVCO Medical Solutions, Orange City, IA). The SFUD, SFIB and IMPT plans used the same two fields: two left anterior oblique superior-inferior fields. For SFIB and IMPT planning, the prescription doses were 64, 60 and 54 Gy(RBE) to the CTV1, CTV 2 and CTV3 in 30 fractions. The corresponding dose to CTV2 and CTV3 for the SFUD plan was 59.7 Gy(RBE) in 28 fractions and 51.2 Gy(RBE) in 24 fractions. The planning target volumes (PTVs) was also defined as 3 mm expansion of the CTVs.

### Conformality and inhomogeneity indices

Isodose distributions were compared visually on axial, sagittal, and coronal slices to assess conformality to the targets and sparing of normal tissues. Target coverage and normal tissue sparing were also compared by dose volume histograms (DVHs). In addition, target coverage was quantitatively assessed by calculation of the conformality index,  and inhomogeneity index  where *PTV* is the planning target volume, *PTV*_*pre*_ is the PTV covered by the prescription dose, and *V*_*pre*_ is the volume of prescription isodose, *D*_*5*_ and *D*_*95*_ are the doses to 5% and 95% of the PTV as displayed on the cumulative DVH, respectively, *D*_*pre*_ is the prescription dose [18]. The larger *COIN* is, the more conformal is the plan. The larger *INH* values indicate a more inhomogeneous dose distribution.

### Comparison of robustness

Robustness of the SFUD, SFIB and IMPT plans with respect to setup and range uncertainties was evaluated using a worst-case analysis method [19]. A setup uncertainty of 3 mm was used for patients 1, 2 and 4 and 5 mm for patient 3. The range uncertainty was assumed to be 3.5% of the nominal ranges [20]. Introduced perturbations included six spatial shifts along the three major axes (left-right, posterior-anterior, and superior-inferior) and two range shifts – for a total of 8 perturbed dose distributions. The highest and lowest dose values in each pixel were extracted from the nominal and the 8 perturbed dose distributions, forming a hot and cold dose distributions with the highest and lowest values, respectively [19]. The width of a banded DVH could then be used to qualitatively represent the robustness of the plans for a specific structure. We selected appropriate dosimetric parameters from the DVHs for the hot and cold plans to compare the robustness of SFUD, SFIB and IMPT plans. For target volumes, *D*_95_ and *D*_98_ for CTVs and generalized equivalent uniform dose (EUD = (Σ*v*_i_*D*_i_^*a*^)^1/*a*^, where *v*_i_ is the fractional organ volume receiving a dose *D*_i_ and *a* is a tissue-specific parameter that describes the volume effect) [14, 21], from the cold plans were used to compare the worst case target coverage. The clinical criteria established in our clinic, are, in the worst case (the cold plan), *D*_95_ ≥ 95%, *D*_98_ ≥ 90% and EUD ≥100% of the prescription dose. For normal tissues, the maximum dose (*D*_max_), mean dose (*D*_mean_) or the percent volume, *V*_*D*_, receiving certain dose, *D*, or more and EUD from hot plans were used to compare the worst case sparing. The EUD parameter *a* was collected from literature [14, 21]: for CTVs, *a* = -10; for spinal cord, *a* = 20; for brainstem, hippocampus, optic chiasm, cochlea, *a* = 16; for femoral heads, *a* = 12; for mandible, *a* = 10; for bladder, rectum *a* = 8; for larynx, *a* = 7.4; for kidney, *a* = 5; and for submandibular gland and whole brain, *a* = 1.

### Statistical analysis

The *COIN, INH*, target coverage in terms of *D*_95_, *D*_98_ and *EUD* in the worst case (cold plans) and normal tissues dosimetric parameters in the worst case (hot plans) were compared statistically by the Wilcoxon matched-pairs signed rank test using GraphPad Prim 6 software (GraphPad Software, Inc., La Jolla, CA). A derived value of *p <* 0.05 was considered statistically significant.

## Results

**Figure 1:**
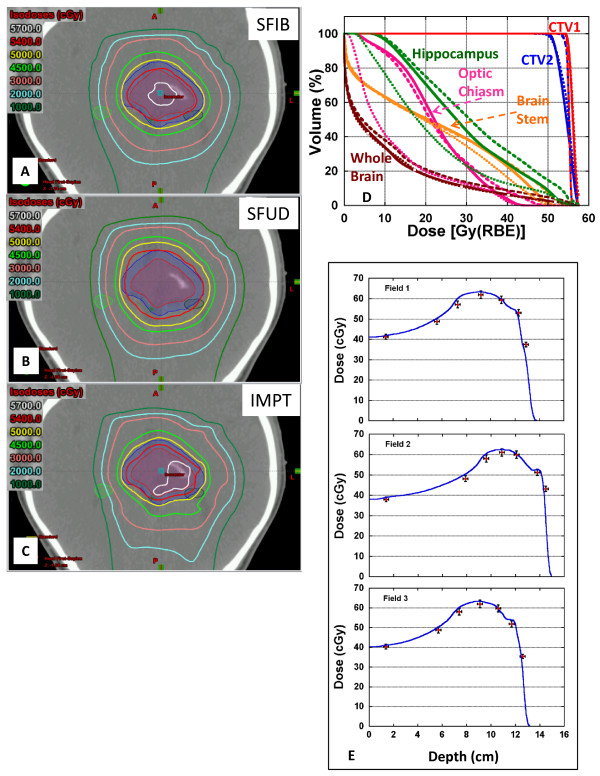
**Representative isodose distributions on axial CT images for patient 1. (A)** SFIB, **(B)** SFUD sequential boost and **(C)** IMPT treatment plans for a central glioma. Targets and normal tissues are represented as color washes. **(D)** Dose volume histogram for corresponding plans. Solid lines correspond to SFIB, dashed lines to SFUD, and dotted lines to IMPT. Exposure of right and left hippocampi to low and high doses is reduced for SFIB and IMPT plans. **(E)** Comparison of measured (data points) and calculated (blue lines) depth dose curves for each of three beams employed in the treatment of patient 1. Error bars are fixed values of 3% in the vertical axis and 2 mm in the horizontal axis to help visualizing the difference between measurements and calculations.

**Figure 2:**
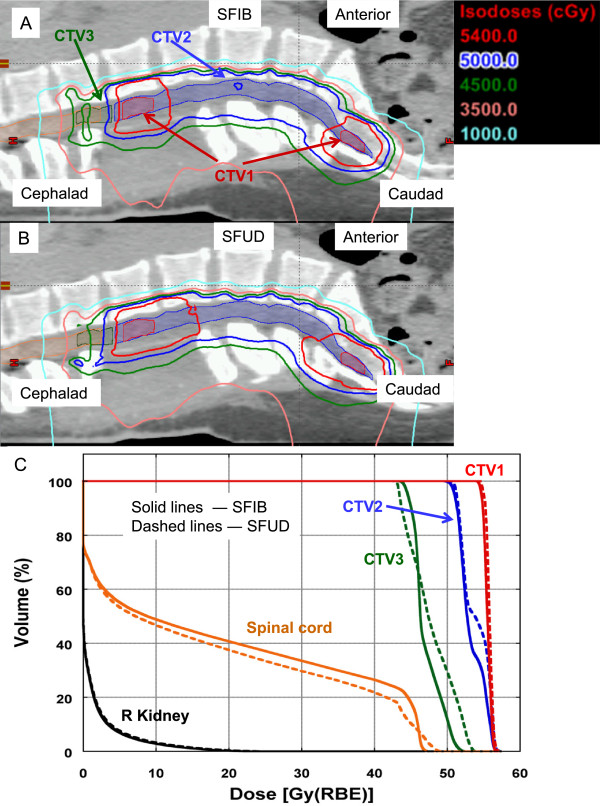
**Representative isodose distributions on sagittal CT images for patient 2. (A)** SFIB and **(B)** SFUD treatment plans for a spinal ependymoma. Targets and normal tissues are represented as color washes. **(C)** Dose volume histogram for corresponding plans (solid lines, SFIB; dashed lines, SFUD).

**Figure 3:**
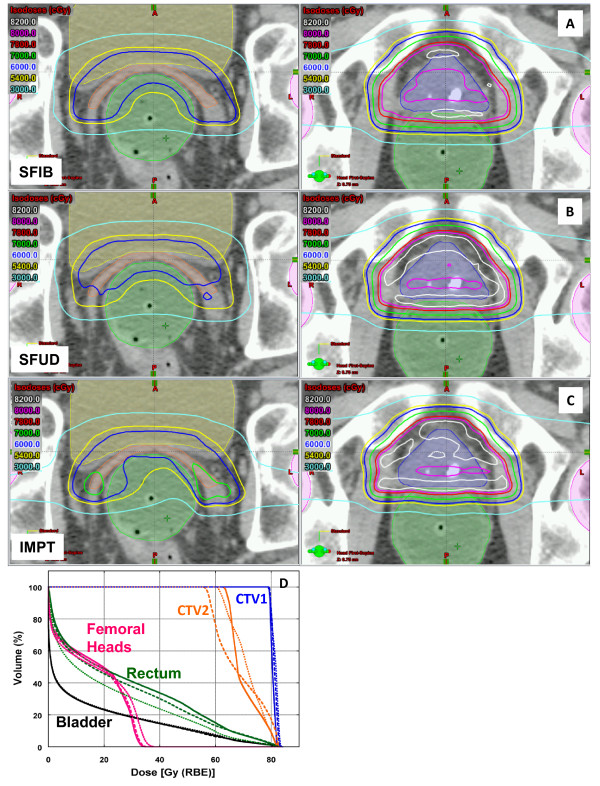
**Representative axial isodose distributions for treatment of a prostatic adenocarcinoma.** Targets and normal tissues are represented as color washes. Slices depicting treatment of CTV2 (seminal vesicles) as well as CTV1 (prostate) are shown in left and right panels for SFIB **(A)**, SFUD with sequential boost **(B)** and IMPT **(C)** plans. The dose volume histogram is shown in **(D)** (solid lines, SFIB; dashed lines, SFUD and dotted lines, IMPT).

**Figure 4:**
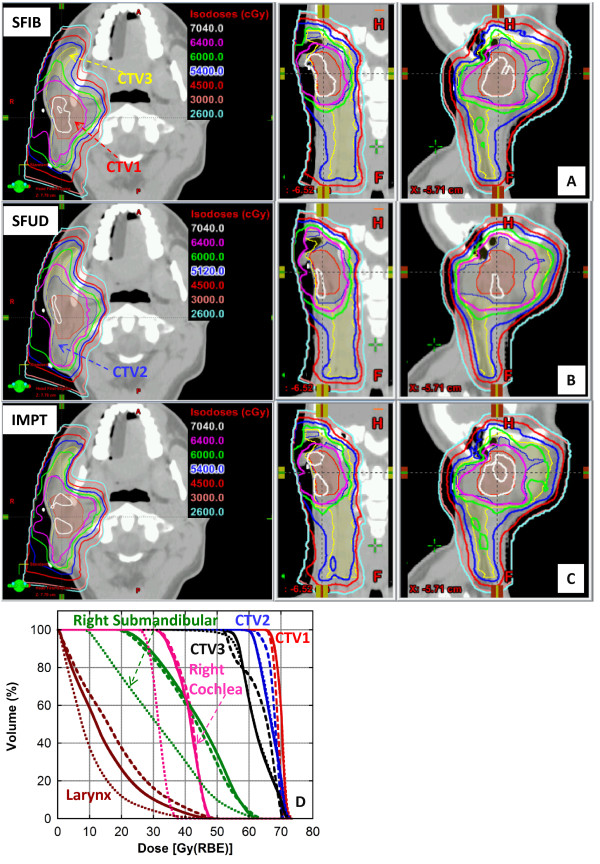
**Representative axial, coronal and sagittal (left, middle, and right panels, respectively) CT images for a patient 4. (A)** SFIB, **(B)** SFUD with sequential boost and **(C)** IMPT treatment plans. Targets volumes are represented as color washes. **(D)** Dose volume histogram (solid lines, SFIB; dashed lines, SFUD and dotted line, IMPT).

Figure 1A-D depicts the isodose distributions and DVHs between the SFIB, SFUD and IMPT plans for the glioma patient. The SFIB planning technique produced a more conformal dose distribution than the SFUD. Moreover, the SFIB technique achieved the desired dose coverage for the target volumes and lower doses to critical normal structures including the right and left hippocampus, and whole brain. The IMPT plan had similar target coverage as SFIB plan, however in this case, normal tissues had similar or less dose. Panel **E** illustrates the calculated and measured depth doses of the SFIB plan. Note that the depth dose curves are not flat as would be a typical curve from a passive scattering or SFUD plan. Instead, we observe a rather non-uniform dose profile, as required by the SFIB technique.For the patient treated for a tumor within the spinal canal, a comparison of isodose distributions and DVHs planned with the SFIB and SFUD technique are shown in Figure 2, respectively and demonstrate that the SFIB plan is qualitatively, more conformal than SFUD plan. Because this patient was treated with a single PA beam, IMPT planning was not applicable.Figure 3 depicts a comparison of the isodose distributions and DVHs between the SFIB, SFUD and IMPT plans for the patient with adenocarcinoma of the prostate. SFIB planning provided a slightly more conformal plan in the region of CTV2 than SFUD. The SFIB technique achieved the desired dose coverage for the target volumes and similar doses to normal structures, including the bladder, rectum, and femoral heads. The IMPT plan had similar target coverage as SFIB plan and a reduced rectum dose but slightly increased femoral head dose.Figure 4 is a comparison of the isodose distributions and DVHs between the SFIB, SFUD and IMPT plans for the head and neck patient. The SFIB technique achieved a conformal dose distribution and met the desired dose coverage for the target volumes while lowering doses to some normal structures, such as the mandible and larynx. The IMPT plans had similar coverage for the target but lower doses to normal tissues.

### Conformality index and inhomogeneity indices

Table 2 lists the *COIN* and *INH* values for PTV1 for SFUD, SFIB and IMPT plans. For all four patients, SFIB plans had larger *COIN* values than SFUD plans (0.585 ± 0.300 vs. 0.435 ± 0.239, Wilconxon test, *p* < 0.01) and thus were more conformal than SFUD plans. However, the difference in *COIN* between SFIB and IMPT was not significant. The inhomogeneity between all three planning techniques was similar (Wilcoxon test, *p* = 0.81).

**Table 2:** **Quantitative comparison of dose conformality and homogeneity**

	COIN			INH		
	SFUD	SFIB	IMPT	SFUD	SFIB	IMPT
**Patient 1**						
**PTV1**	0.507	0.867	0.827	0.021	0.063	0.058
**PTV2**	0.422	0.857	0.893	-	-	
**Patient 2**						
**PTV1**	0.771	0.771	-	0.066	0.056	-
**PTV2**	0.539	0.577	-	-	-	-
**PTV3**	0.026	0.033	-	-	-	-
**Patient 3**						
**PTV1**	0.859	0.910	0.851	0.043	0.038	0.057
**PTV2**	0.140	0.159	0.154	-	-	
**Patient 4**						
**PTV1**	0.273	0.427	0.453	0.062	0.089	0.091
**PTV2**	0.474	0.613	0.638	-	-	
**PTV3**	0.608	0.633	0.613	-	-	
**Mean ± SD (All patients)**	0.435 ± 0.239*	0.585 ± 0.300*	-	0.048 ± 0.021	0.062 ± 0.021	-
**Mean ± SD (exclude patient 2)**	0.469 ± 0.232	0.638 ± 0.274	0.633 ± 0.263	0.042 ± 0.021	0.063 ± 0.026	0.069 ± 0.019

*Abbreviations*: *COIN* conformal index, *INH* inhomogeneity index, *PTV* planning target volume, *SFIB* single-field integrated boost, *SFUD* single field uniform dose, *IMPT* intensity modulated proton therapy, *SD* standard deviation. *p < 0.01, Wilcoxon matched-pairs signed rank test.

### Robustness evaluation

**Figure 5:**
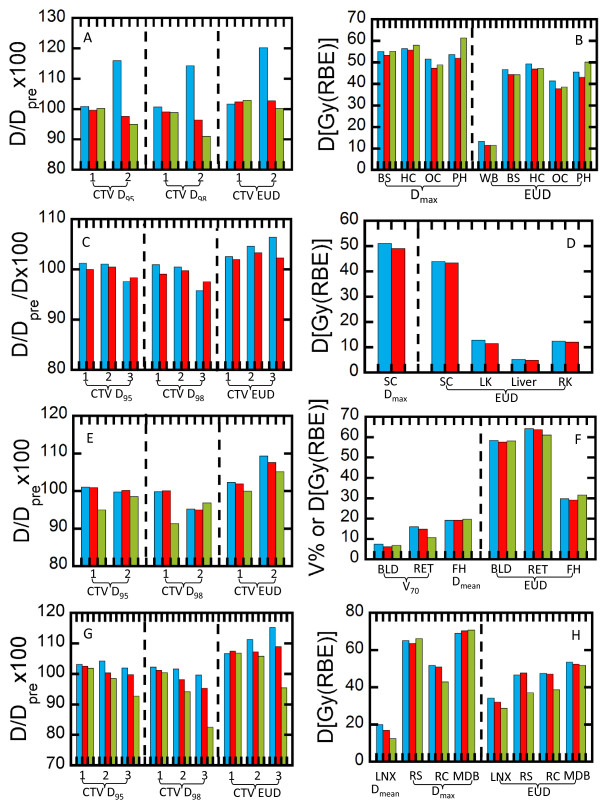
**Worst case robustness comparison of SFUD, SFIB and IMPT/MFO plans.** The left column (blue) represents SFUD, the middle (red), SFIB, and the right (green), IMPT plans. For target volumes, the cold plans are used to evaluate target coverage in terms of percentage of doses of D_95_, D_98_ and EUD relative to the prescription doses. For critical structures, the hot plans are used assess the tissue sparing. **(A)** and **(B)** for patient 1 (BS – brain stem, HC – hippocampus, OC – optical chiasm, PH – pituitary and hypothalamus, WB – whole brain); **(C)** and **(D)** for patient 2 (SC – spinal cord, LK – left kidney, RK – right kidney); **(E)** and **(F)** for patient 3 (BLD – bladder, RET – rectum, FH – femoral heads); and **(G)** and **(H)** for patient 4 (LNX- larynx, RS – right submandibular, RC – right cochlea, and MDB – mandible).

Shown in Figure 5 is a summary of the comparison of worst case robustness of SFUD, SFIB and IMPT plans in terms of both target coverage and normal tissue sparing in column bar plots. For target coverage of all 4 patients, *D*_95_, *D*_98_ and EUD for the SFIB plans were smaller than SFUD plans in the worst case, cold plans, Table 3 (mean ± standard deviation, *D*_95_ = 100.0 ± 1.3 vs. 102.7 ± 5.0, *D*_98_ = 98.1 ± 2.1 vs. 101.1 ± 5.2, EUD = 104.6 ± 2.8 vs. 108.0 ± 6.1, SFIB vs. SFUD, respectively, Wilcoxon test, p < 0.05 for *D*_95_, *D*_98_, and EUD). This is consistent with SFIB plan being more conformal than SFUD plans. Despite the slightly smaller values of *D*_95_, *D*_98_ and EUD, all SFIB plans meet the clinical criteria established in our clinic, that is, in the worst case (the cold plan), *D*_95_ ≥ 95%, *D*_98_ ≥ 90% and EUD ≥100% of the prescription dose (Table 3).

**Table 3:** **Summary of the worst-case target coverage as percentage of prescription dose**

	SFUD% (range)	SFIB% (range)	IMPT% (range)
**D**_**95**_ **/D**_**pre**_ **(All patients)**	102.7 ± 5.0	100 ± 1.3	-
	(97.6-115.9)	(97.6-102.5)	-
**D**_**98**_ **/D**_**pre**_ **(All patients)**	101.1 ± 5.2	98.1 ± 2.1	-
	(95.2-114.2)	(95.0-101.2)	-
**EUD/D**_**pre**_ **(All patients)**	108.0 ± 6.1	104.6 ± 2.8	-
	(101.6-120.2)	(101.9-108.9)	-
**D**_**95**_ **/D**_**pre**_ **(exclude patient 2)**	103.8 ± 5.5	100.1 ± 1.5	97.4 ± 3.3
	(99.7-115.9)	(97.6-102.5)	(92.7-101.9)
**D**_**98**_ **/D**_**pre**_ **(exclude patient 2)**	101.9 ± 5.9	97.9 ± 2.4	93.6 ± 6.1
	(95.2-114.2)	(95.0-101.2)	(82.4-100.4)
**EUD/D**_**pre**_ **(exclude patient 2)**	109.5 ± 6.7	105.5 ± 3.0	102.3 ± 4.1
	(101.6-120.2)	(101.9-108.9)	(95.4-106.8)

*Abbreviations:* *D*_*95*_ and *D*_*98*_, doses to 95% and 98% of the target volumes as displayed on the cumulative DVH, respectively, *D*_*pre*_, prescription dose, *EUD* equivalent uniform dose, *SFUD* single field uniform dose, *SFIB* single-field integrated boost, *IMPT* intensity modulated proton therapy.

For target coverage of the three cases with IMPT plans, *D*_95_, *D*_98_ and EUD for the IMPT plans were smaller than SFIB plans in the worst case, cold plans, Table 3 (mean ± standard deviation, *D*_95_ = 97.4 ± 3.3 vs. 101.1 ± 1.5, *D*_98_ = 93.6 ± 6.1 vs. 97.9 ± 2.4, EUD = 102.3 ± 4.1 vs. 105.5 ± 3.0, IMPT vs. SFIB, respectively, Wilcoxon test, p < 0.05 for *D*_95_ and EUD, and p = 0.078 for *D*_98_). However, some IMPT plans failed to meet the clinical criteria in the worst case scenario (the cold plans), with minimum values of *D*_95_, *D*_98_ and EUD of 92.7%, 82.4% and 95.4% of the prescription doses respectively, values below our clinical criteria (Table 3).For normal tissues, SFIB was significant better than SFUD even in the worst case (the hot plans) (dosimetric parameters and EUDs in Figure 5, SFIB vs. SFUD, Wilcoxon test, p < 0.01). One noted exception was the mandible for patient 4 as presented in Figure 5H. This exception is likely due to the fact that the mandible overlapped with target volume in this case. On the other hand, the difference between IMPT and SFIB in terms of normal tissues in the worst cases (hot plans) was insignificant (Wilcoxon test, p = 0.58 and 0.45 for dosimetric parameters and EUDs, respectively).

## Discussion

SSPT employing an SFIB approach is commonly used in our clinical practice. Here, we have presented examples of treatment plans utilizing the SFIB technique. We found in each disease site, the SFIB technique generated more conformal dose distributions than the SFUD technique. Moreover, SFIB plans were robust in the face of uncertainties. For target coverage, all SFIB plans meet clinical criteria even in the worst-case scenario; for normal tissues, the SFIB plans were most often superior to SFUD plans with the exception cases where normal tissues of concern overlapped with the target volume. Our clinical experience suggests that not only does SFIB result in superior treatment plans but also expedites treatment planning and patient specific QA.

**Figure 6:**
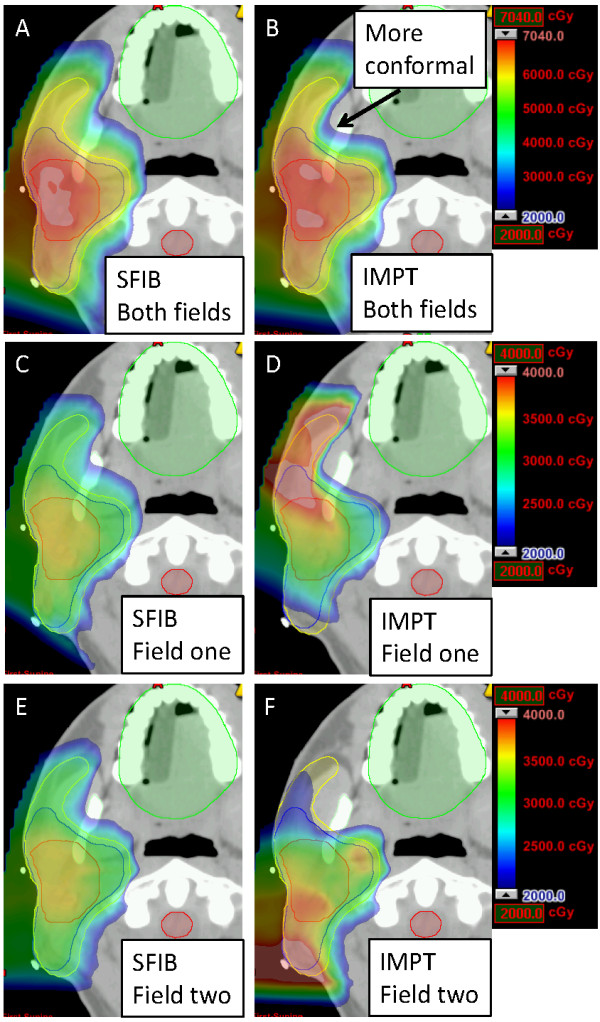
**Example of dose distributions of SFIB and IMPT for patient 4 in color wash to demonstrate the dose inhomogeneity of individual fields of an IMPT plan. (A)** SFIB and **(B)** IMPT for all fields; **(C)** SFIB and **(D)** IMPT for field one; and **(E)** SFIB and **(F)** IMPT for field two.

SSPT offers exciting and challenging new opportunities, such as IMPT. In IMPT, the dose for each individual field is highly inhomogeneous within each target volume. When all fields are combined, the total doses would result in the desired dose distribution for all target volumes. Shown in Figure 6 are the IMPT plan dose distributions from combined and individual fields for patient 4. There is little difference between SFIB and IMPT for the combined dose distributions (Figure 6A and B), except the IMPT dose distribution is more conformal in certain areas (arrow, Figure 6B). For both field one or two, the IMPT plan shows much more inhomogenous dose distribution than the SFIB plan. One could consider IMPT as a sophisticated version of the patch/match technique, sometimes used in passively scattered proton beam therapy planning [1]. IMPT may produce more conformal dose distributions, but may also be more sensitive to various uncertainties, including setup and range uncertainties.

In the current work, worst case robust analysis showed that IMPT were less robust than SFIB plans in terms of target coverage. It should be pointed out that the optimization phase space of a SFIB plan is a subset of that of IMPT. In other words, it is possible, in theory, to obtain a solution of SFIB with MFO. In practice, however, one would never arrive at a solution of SFIB using MFO due to very large number of degenerated solutions available for MFO. In our clinical practice, we have also found that the robustness of an IMPT plan is optimization technique dependent; that is, IMPT plans generated by some initial conditions are more robust than others. For the examples presented here, the SFIB technique achieved the similar dose conformity and normal tissue sparing to IMPT and yet provided some assurance of robustness of the plan.

The SFIB technique may not be ideally applied to all cancer patients who received SSPT and require multiple dose levels. For complex target volumes such as with oropharyngeal and nasopharyngeal tumors requiring treatment of the bilateral neck, it is recommended to use IMPT as the planning method given the complex geometry of the targets [19]. Indeed robust optimization, considering various uncertainties, is recommended for general IMPT planning [6, 7, 22].

The SFUD technique produces a flat depth dose profile within the target along the beam direction by the superposition of many spots with different energies. However, a flat depth dose profile is not required with SFO. In fact, the planner has all the degrees of freedom to produce a non-uniform dose distribution along the beam and lateral directions with SFO. SFO is a more general concept than SFUD and may be used to generate either SFUD or SFIB plans on a field-by-field basis. It should be noted that identical beams were used for SFUD primary and boost plans in this work. If different beam angles were used for the boost SFUD plans, the normal tissues might be better spared. However, this was not explored in the current study.

In a planning study, Cozzia *et al.* proposed an SIB technique based on SFUD for head and neck cancer patients with two target volumes and two prescription dose levels [23]. They optimized fields for each target individually and then summed them to obtain the final plan. In contrast to the SFUD-based SIB technique by Cozzia *et al.* [23], the SFIB method presented in this work is an integrated approach in terms of both treatment planning and delivery.

Compared with the SFUD sequential boost approach, SFIB offers seamless delivery and more efficient treatment planning and patient-specific QA (one plan for SFIB vs. two or three plans for sequential boost SFUD). We estimate that there is a 30% to 50% time saving in both treatment planning and patient specific QA with the SFIB technique. This increased efficiency provides additional logistical and economic reasons for treating patients with SFIB. Currently, proton therapy, especially SSPT, is a limited resource available in the United States and around the world. As the SSPT treatment process becomes more efficient, more patients may benefit from this advanced form of radiation therapy.

The plans presented in this work used specific beam settings available in our clinic, as detailed in Table 1. If the beam settings were significantly different from these in Table 1, some of the specific results might be different. For example, if the spot size was much smaller, the spot spacing should be smaller to keep the ratio spot spacing to spot size in air ≤ 1.5. Then the conformal index would be improved for all plans yet the robustness would be reduced with respect to the setup uncertainties due to sharper penumbra associated with smaller spot size. However, one should expect that the SFIB still is a valid planning technique to generate conformal and robust plans for selected patients with relatively simple targets.

## Conclusion

We have successfully implemented an SFIB technique based on SFO for SSPT to treat a variety of cancer types. SFIB is a natural application of SFO. When SFO is used, a uniform dose across the target volume is not required. Therefore, SFO is a general concept and can be used to generate either SFUD or SFIB plans. SFIB often produces more conformal plans compared to those with SFUD. Additional advantages of SFIB are seamless delivery and more efficient treatment planning and patient-specific QA. For relatively simple target volumes presented in this work, we also demonstrated that SFIB may be more robust than IMPT, yet has similar conformity and normal tissue sparing.
